# Supplementary material for: MetaRibo-Seq measures translation in microbiomes
Source: Nat Commun. 2020 Jun 29;11:3268. doi: 10.1038/s41467-020-17081-z (PMC7324362; doi:10.1038/s41467-020-17081-z)
Supplement: Supplementary file 10 — Supplementary Data 7 [file 41467_2020_17081_MOESM10_ESM.zip › File2/Confidence_High_Taxonomy/57080_out.krona.html]

Javascript must be enabled to view this page.

members
magnitude
magnitudeUnassigned
count
unassigned
taxon
rank

57080\_out

60

60
2
superkingdom

1
976
phylum

1
class
200643

171549
order
1

1
family
815

1
genus
816

1
species
387090

SRS147425\_contig\_number\_220

59
phylum
1239

class
186801
59

59
order
186802

541000
family
59

59
3

SRS053214\_contig\_number\_contig-100\_32568.32569SRS063489\_contig\_number\_contig-100\_20882.20882SRS1055043\_contig\_number\_contig-100\_30174.66867
1263
genus

1160721

SRS014855\_contig\_number\_11772SRS077294\_contig\_number\_16323SRS143523\_contig\_number\_25038SRS893252\_contig\_number\_13702
species
4

species

SRS011084\_contig\_number\_32038SRS013940\_contig\_number\_contig-100\_169.139670SRS014235\_contig\_number\_44276SRS014979\_contig\_number\_19325SRS015578\_contig\_number\_18794SRS015854\_contig\_number\_2977SRS016018\_contig\_number\_contig-100\_7990.67172SRS016495\_contig\_number\_contig-100\_7466.80784SRS016954\_contig\_number\_14605SRS018541\_contig\_number\_13030SRS019685\_contig\_number\_41734SRS020394\_contig\_number\_14891SRS022071\_contig\_number\_16870SRS024625\_contig\_number\_contig-100\_263.120078SRS043768\_contig\_number\_10304SRS044535\_contig\_number\_contig-100\_3488.3489SRS045826\_contig\_number\_2582SRS065504\_contig\_number\_18854SRS075716\_contig\_number\_2392SRS098073\_contig\_number\_2327SRS1041038\_contig\_number\_14418SRS1041157\_contig\_number\_6110SRS105082\_contig\_number\_contig-100\_25746.25747SRS142781\_contig\_number\_11529SRS148159\_contig\_number\_52835SRS893358\_contig\_number\_13715
41978
26

7

SRS012273\_contig\_number\_12548SRS018817\_contig\_number\_21518SRS019161\_contig\_number\_contig-100\_1972.127507SRS019178\_contig\_number\_4732SRS054352\_contig\_number\_17870SRS142980\_contig\_number\_6737SRS150029\_contig\_number\_17500
2293149
species

11
165186

SRS013638\_contig\_number\_11962SRS015694\_contig\_number\_10864SRS023914\_contig\_number\_14463SRS023914\_contig\_number\_15128SRS048164\_contig\_number\_19667SRS049446\_contig\_number\_14387SRS053335\_contig\_number\_13072SRS1055067\_contig\_number\_946SRS140513\_contig\_number\_contig-100\_3031.3031SRS142503\_contig\_number\_23256SRS149325\_contig\_number\_10467
species

1
species
2293161

SRS147766\_contig\_number\_contig-100\_3976.133896

1
species
2293165

SRS018351\_contig\_number\_15664


SRS011302\_contig\_number\_29203
2293156
species
1

4
species
2293194

SRS016629\_contig\_number\_3436SRS042628\_contig\_number\_4885SRS104636\_contig\_number\_27518SRS893300\_contig\_number\_9512

1
species
1637508

SRS076804\_contig\_number\_18226
